# What are parents’ priorities when being invited to enter their child into a clinical trial and how far do they voice their priorities?

**DOI:** 10.1186/1745-6215-14-S1-O72

**Published:** 2013-11-29

**Authors:** Bridget Young, Kerry Woolfall, Paula Williamson

**Affiliations:** 1 University of Liverpool, Liverpool, UK

## Objectives

Research has tended to focus on the information that researchers and ethicists deem important for informed consent to clinical trial participation and on the deficits in patients’ understanding of this information. Drawing on an alternative ‘capabilities’ approach we explored what information parents prioritised when making a decision about their child’s participation in a clinical trial, parents’ comprehension of these issues and how far they voiced their priorities during discussions with recruiters.

## Methods

Qualitative interview and observational study examining recruitment in four placebo-controlled, double-blind randomised clinical trials of medicines for children. We compared audio-recorded routine parent-recruiter discussions (N=41) about the clinical trials with subsequent semi-structured interviews with parents about their experiences of recruitment.

## Results

When making a decision about trial entry, parents’ main priorities were: what clinical benefit the trial might offer their child, their child’s safety; practicalities of participation; research for the common good: access to medication and randomisation. Parents had specific misunderstandings linked to these priorities which had the potential to influence their decisions. While parents had many questions and concerns about trial participation which influenced their decision-making, they rarely voiced these during discussions with recruiters.

## Conclusions

Knowing what information patients and carers prioritise when deliberating about entry to a trial can help recruiters when approaching potential participants. Tailoring discussion around these issues and helping patients to voice their particular priorities may enhance recruitment conduct.

